# Influence of pure fluorides and stannous ions on the initial bacterial colonization *in situ*

**DOI:** 10.1038/s41598-019-55083-0

**Published:** 2019-12-06

**Authors:** Kirsch Jasmin, Hannig Matthias, Winkel Pia, Basche Sabine, Leis Birgit, Pütz Norbert, Kensche Anna, Hannig Christian

**Affiliations:** 1 Clinic of Operative Dentistry, Medical Faculty Carl Gustav Carus, Technische Universität Dresden, Fetscherstraße 74, D-01307 Dresden, Germany; 2 Clinic of Operative Dentistry, Periodontology and Preventive Dentistry, University Hospital, Saarland University, Building 73, D-66421 Homburg, Saar Germany

**Keywords:** Dental biofilms, Fluoridation

## Abstract

The present clinical-experimental study aims to examine the effect of pure experimental fluoride solutions and stannous chloride on the initial oral bioadhesion under *in situ* conditions. After 1 min of pellicle formation on bovine enamel slabs, 12 subjects rinsed with 8 ml of the fluoride test solutions (NaF, Na_2_PO_3_F, AmF, SnF_2_,) with 500 ppm fluoride concentration each for 1 min. Additionally, rinsing without a solution (control) and rinsing with 1563 ppm SnCl_2_ solution took place for 1 min. Afterwards, fluorescence microscopy took place to visualize bacterial adhesion and glucan formation (8 h oral exposition) with DAPI and ConA and the BacLight method. TEM was performed to visualize the pellicle ultrastructure together with EDX to detect stannous ions. The rinsing solutions with pure SnF_2_ and SnCl_2_ reduced significantly the initial bacterial colonization (DAPI). While, NaF and Na_2_PO_3_F showed no significant effect compared to the control. There was no significant difference between AmF, SnF_2_ and SnCl_2_. All tested experimental solutions showed no reducing effect on the glucan formation. Considerable alterations of the pellicle ultrastructure resulted from rinsing with the Sn-containing solutions. SnF_2_ appears to be the most effective type of fluoride to reduce initial bacterial colonization *in situ*. The observed effects primarily have to be attributed to the stannous ions’ content.

## Introduction

According to the WHO oral health report of october 2017, dental caries is still a major public health problem and the most common non transmittable disease worldwide, especially in industrialized countries affecting 60–90% of the school children and a vast majority of adults. It is expensive to treat; hence consuming 5–10% of healthcare budgets and is the main reason for hospitalization of children in some high-income countries. In this context, the use of fluorides is recommended worldwide. In order to cite the WHO: “It is important that population-wide prevention interventions are universally available and accessible. Such interventions include the use of fluoride, and comprehensive patient-centred essential oral health care”. This quote emphasizes the use and application of fluorides in daily dental health care. It is even more important that the mechanisms, effects and actions of fluorides are fully understood, especially when the use is recommended worldwide on a daily basis. Nevertheless, there are different explanations how fluorides work. Current explanatory models are based on studies with combinations of fluorides or fluoride solutions with different amounts of constituents. Thereby, many studies on the anticariogenic effect of fluorides focused on their role in reducing enamel and dentin demineralization and enhancing remineralization of early caries lesions^1,2^. Multiple clinical studies demonstrated the cariostatic effect of fluorides in various multifaceted application forms.

Initial biofilm formation starts with the adhesion of oral microorganisms to the pellicle layer, a physiological proeinaceous layer with protective and antibacterial properties^3,4^. The adhesion of singular bacterial cells is followed by coaggregation and bacterial cell multiplication as well as glucan synthesis^5,6^. Despite the well known efficacy of fluorides, little is known about the interactions of pure fluoride substances with the pellicle layer and the effect of these substances on initial bioadhesion as the initial point of biofilm formation under *in situ* conditions^7^. In contrast, fluoride compounds in already existing mouth rinsing solutions or tooth pastes are the objective of multiple clinical and experimental trials^7^. These mouth rinses have a high variety of constituents and combinations of fluoride components^7,8^. Thereby, chemical interactions of the constituents are possible and the antiadherent effect might be influenced by specific components of the mouthrinses.

In this context, *in vitro* research showed, that fluoride concentrations above 300 ppm can significantly reduce acidogenicity, acidurity and the development of three dimensional bacterial aggregates of streprococcus mutrans communities^9^. In addition, it is known that fluorides can inhibit the bacterial enzymes^9^.

Previous studies and reports have shown that especially the metal cations of fluorides play an important role in prevention and treatment of enamel erosions^10,11^. Thereby, significant differences between the cations were revealed for their anti-erosive effect^10,11^ So far, there is little evidence of their influence in initial oral bioadhesion processes. An earlier *in vitro* study has shown, that sodium fluoride was superior to amine fluoride concerning its anti-erosive capacity^10^. Stannous ions in stannous fluorides can inhibit the bacterial enzymes aldolase and glycerinaldehyde dehydrogenase by oxidation of thiol-groups. As a consequence, the bacterial metabolism is negatively influenced. In addition, the bacterial cell-cell-cohesion and adhesion to enamel surfaces are inhibited by stannous ions. This can lead to an antiadherent effect induced by these metal ions^12^. These results are very important findings supporting an increase of knowledge of the actions and mechanisms of fluorides. Nevertheless, to our best knowledge there is no *in situ* study that has examined the effect of pure fluorides (sodium fluorophosphates, sodium fluoride, amine fluoride, stannous fluoride) on initial oral bioadhesion processes and the modifying action on the ultrastructure.

## Results

**Figure 1:**
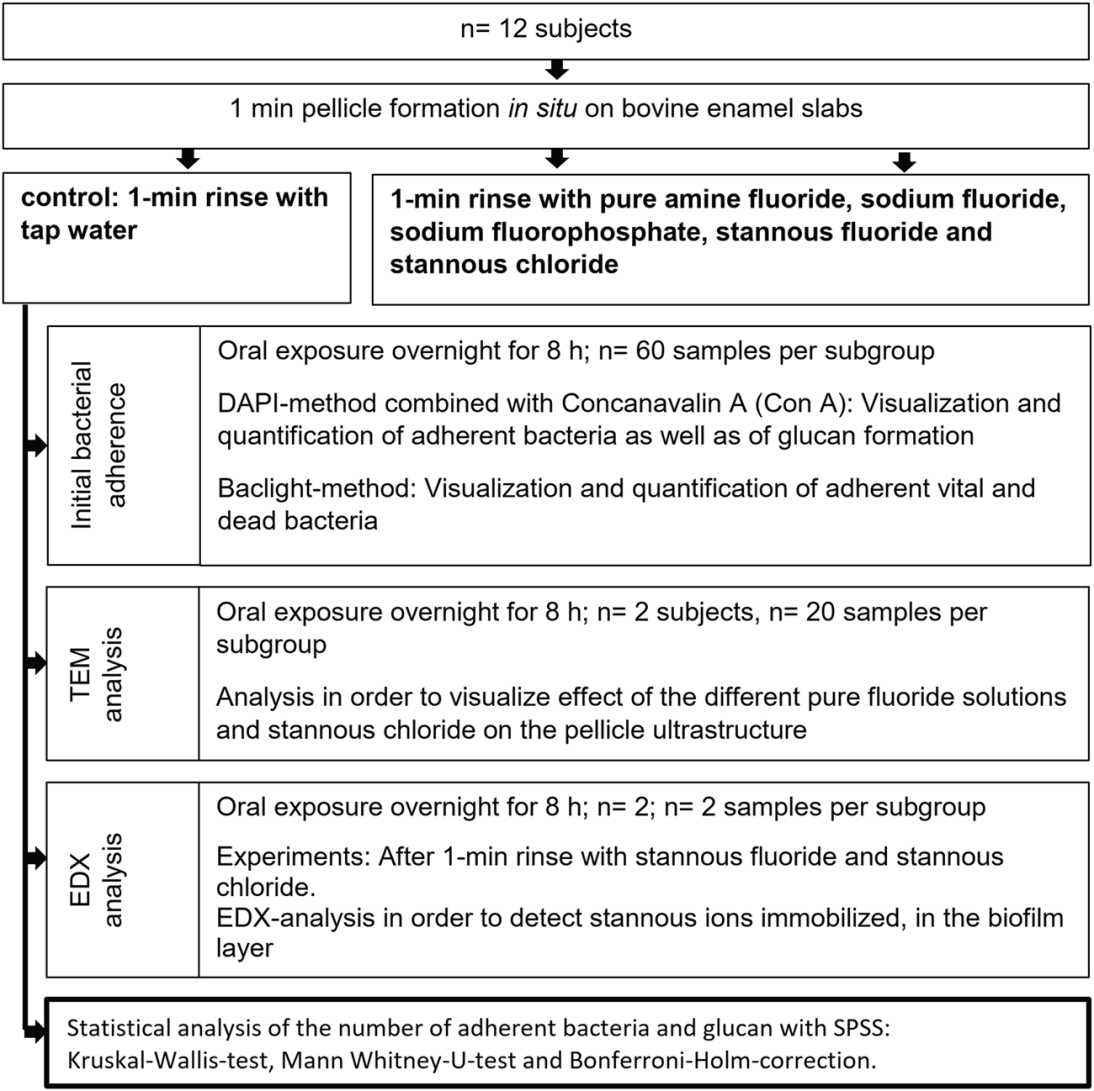
Flow chart of the *in situ* experiments. Thereby, every volunteer (n = 12) rinsed with the different fluoride solutions and the stannous chloride solution at different days (2-day wash out period between the different experiments). After oral overnight exposure for 8 h, the specimens were removed and used for the experiments (DAPI, BacLight) irrespective of their position on the splint. The experiments for TEM and EDX analysis took place with selected subjects (n = 2). The statistical analysis was performed for the number of adherent bacteria and glucans with the Kruskal-Wallis-test, the Mann Whitney-U-test and the Bonferroni-Holm-correction.

The bovine specimens were analysed with DAPI, BacLight, SEM and EDX-analysis (Fig. 1).

**Figure 2:**
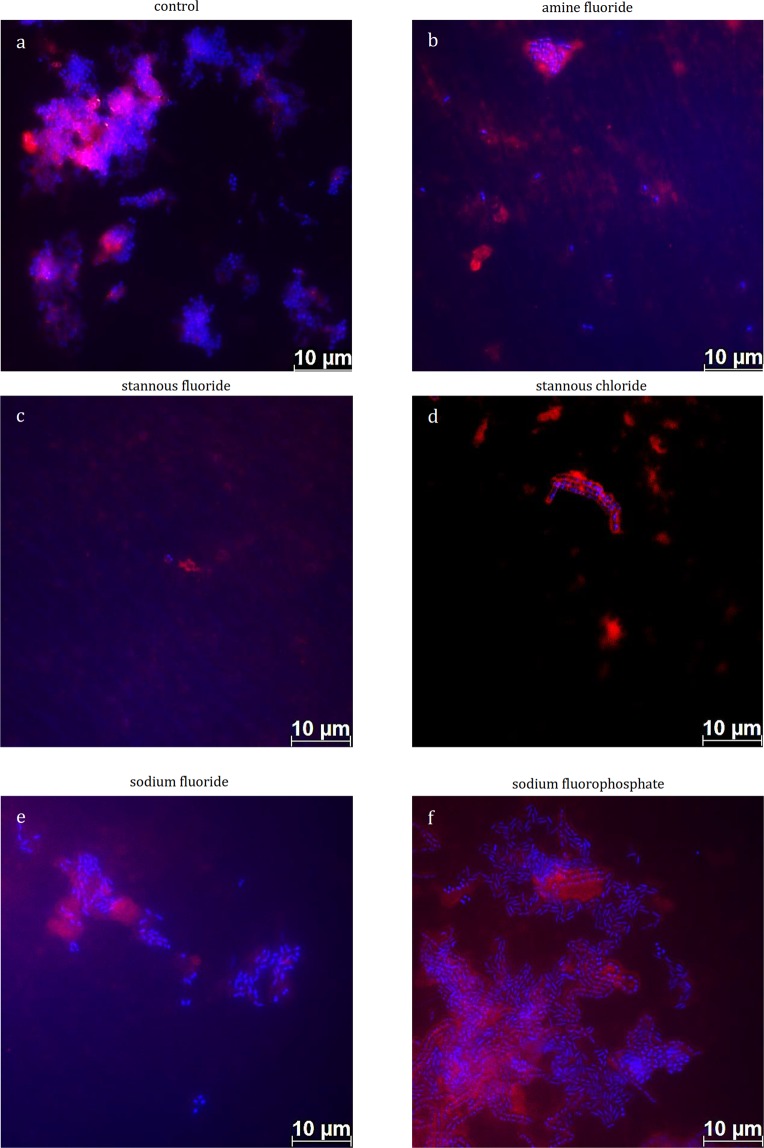
Fluorescence microscopic images displaying a combination of simultaneous DAPI (blue) and glucan (red) staining. Thereby DAPI-staining indicates adherent bacteria at the pellicle layer as well as bacteria-glucan-agglomerates in combination with the glucan staining (Con A – red). The highest number of adhering bacteria is detected after 1-min-rinsing with tap water (**a**, control), sodiumfluoride (**e**) and sodium fluorophosphate (**f**). In contrast, less adherent bacteria are stained after 1-min-rinsing with amine fluoride (**b**), stannous fluoride (**c**) and stannous chloride (**d**). *In situ* pellicle formation time: 8 h.

**Figure 3:**
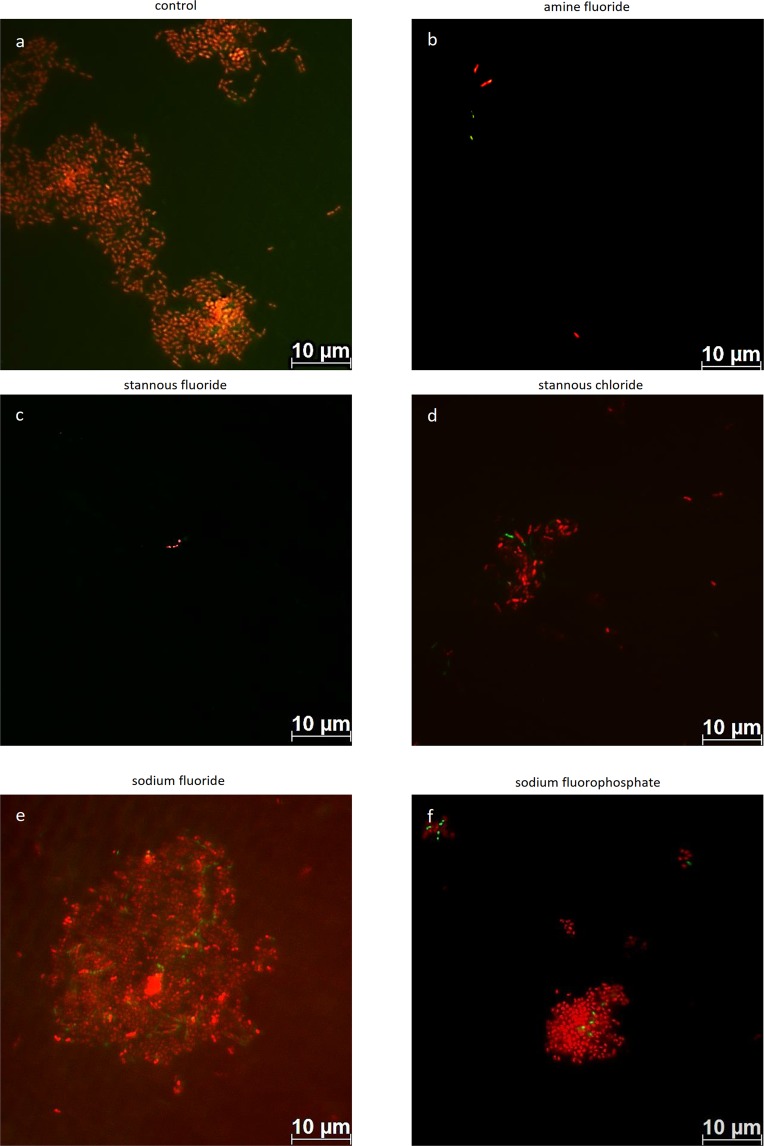
Fluorescence microscopic images after staining with the Live/dead staining BacLight kit. Thereby, vital bacteria (green) and dead bacteria (red) are detectable at the pellicle layer. The highest number of vital and dead bacteria is detected after 1-min-rinsing with tap water (**a**, control), sodiumfluoride (**e**) and sodium fluorophosphate (**f**). In contrast, less adherent bacteria are stained after 1-min-rinsing with amine fluoride (**b**), stannous fluoride (**c**) and stannous chloride (**d**). *In situ* pellicle formation time: 8 h.

Initial bacterial colonization was thereby visualized by DAPI (Fig. 2) and BacLight staining (Fig. 3). On the enamel slabs of the control group, monolayers of adherent bacteria were visible covering more than 50% of the enamel surface (Fig. 2a). The rinsing solutions with pure stannous fluoride (Fig. 2c) and stannous chloride (Fig. 2d) significantly reduced the initial bacterial colonization *in situ*. Thereby single bacterial cells, and small aggregates were detectable on enamel (Fig. 2c,d). In addition, the visualization of the glucan formation took place with the fluorescence dye Concanavalin A (Fig. 2). In general, all tested experimental solutions showed no reducing effect on the glucan formation (Fig. 2a–f). Thereby, glucan formation was detectable on all enamel samples, in particular, around adhering bacterial cells (Fig. 2d).

With the BacLight live/dead staining a clear differentiation of vital (green) and dead (red) bacteria was possible (Fig. 3). Thereby, less vital bacteria were detectable after rinsing with amine fluoride (Fig. 3b) and stannous fluoride (Fig. 3c) compared to the control (Fig. 3a) and the sodium fluoride group (Fig. 3e). In addition less adherent dead bacteria were visible on the enamel surface after rinsing with amine fluoride (Fig. 3b) and stannous fluoride (Fig. 3c) compared to the control (Fig. 3a) and sodium fluoride group (Fig. 3e). Further, significantly fewer dead bacteria were detectable after the rinse with stannous fluoride (Fig. 3b) and amine fluoride (Fig. 3b) compared to sodium fluorophosphate (Fig. 3f).

**Figure 4:**
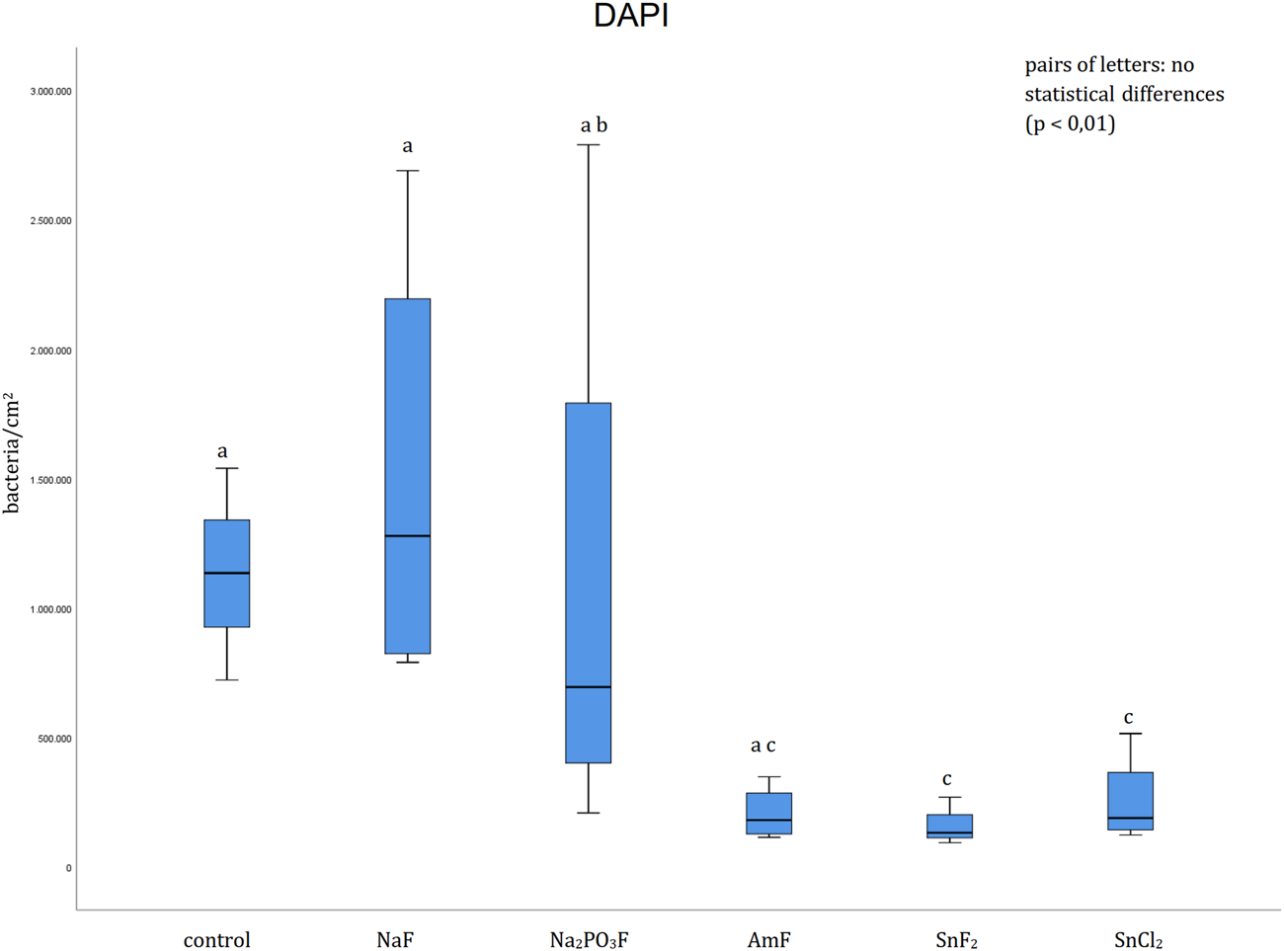
Boxplot diagram. Influence of pure fluoride solutions on initial bacterial adhesion to enamel bovine slabs *in situ*. Evaluation of the bacterial colonization with the DAPI method. Columns sharing the same letter (enamel: lower case letters) are not significantly different (Mann-Whitney U test and Bonferroni-Holm correction, p < 0.01).

A quantification of the adherent bacteria was performed after visualization of the bacterial cells with DAPI (Fig. 4). In the control group, the participants rinsed with tap water and significantly higher numbers were detected on enamel compared to stannous fluoride and stannous chloride_._ In contrast, amine fluoride, sodium fluoride and sodium fluorophosphate showed no significant effect compared to the control. After rinsing with sodium fluoride significantly more adherent bacteria were detected compared to stannous fluoride and stannous chloride after visualization with DAPI. In addition, significantly fewer bacteria were visible after the rinse with stannous fluoride compared to sodium fluorophosphate. Besides, no significant difference was revealed by the DAPI method between the rinsing solutions amine fluoride, stannous fluoride and stannous chloride (Fig. 4).

**Figure 5:**
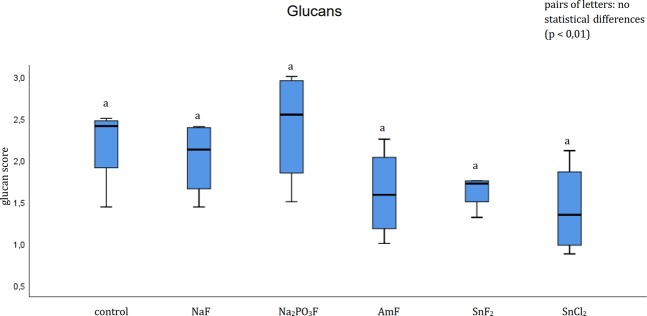
Boxplot diagram. Influence of pure fluoride solutions on glucan formation on enamel bovine slabs *in situ*, staining dye Concanavalin A. Columns sharing the same letter (lower case letters) are not significantly different (Mann-Whitney U test and Bonferroni- Holm correction, p < 0.01), in summary no statistical differences were shown.

The evaluation of the glucan formation showed no significant effect of all pure fluoride solutions and stannous chloride (Fig. 5).

**Figure 6:**
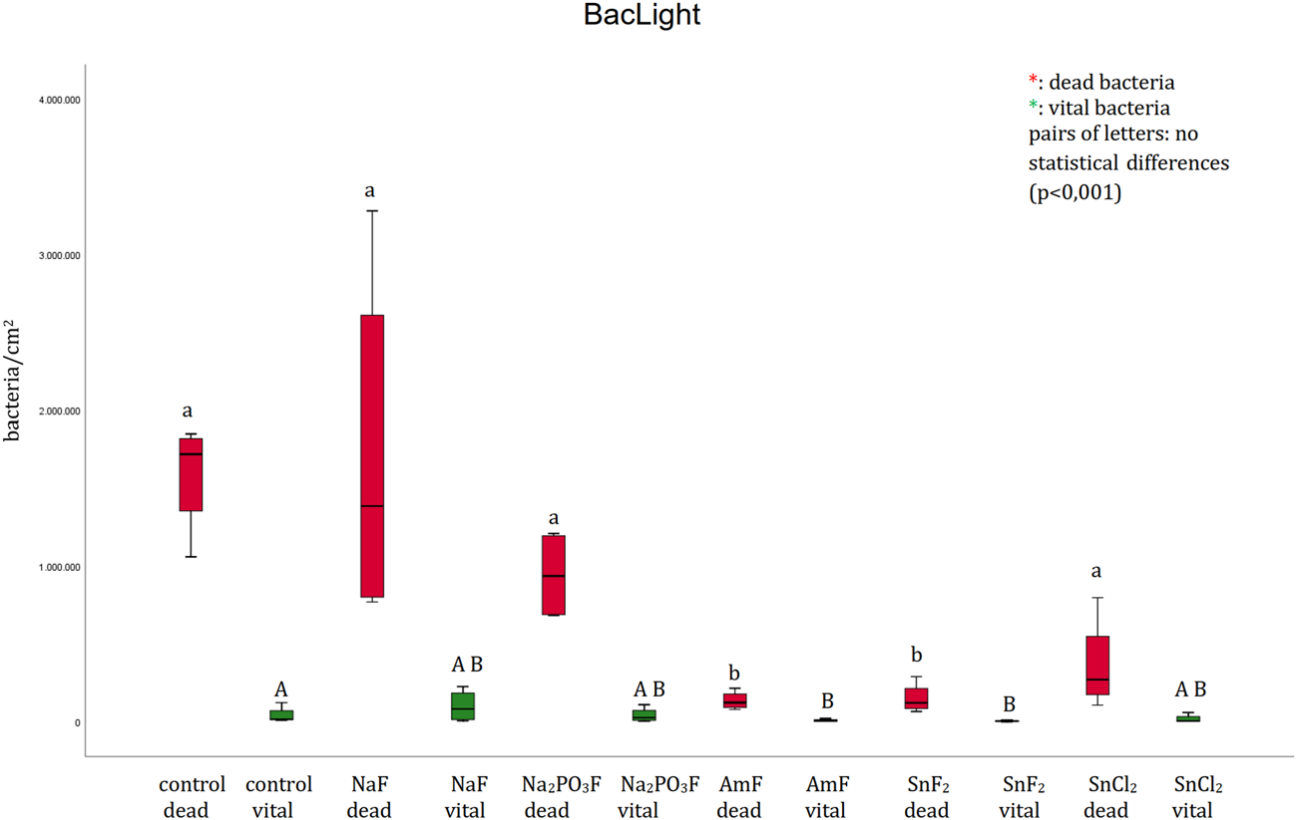
Boxplot diagram. Influence of pure fluoride solutions on initial bacterial adhesion to enamel bovine slabs *in situ*. Evaluation of vital (green) and dead (red) bacteria with the BacLight method. Columns sharing the same letter (enamel: lower case letters) are not significantly different (Mann-Whitney U test, and Bonferroni-Holm correction, p < 0.01). Significantly lower numbers of bacteria are detectable after rinsing with amine fluoride (vital and dead bacteria) and stannous fluoride (vital and dead bacteria) compared to the control (rinsed with tap water), sodium fluoride and sodium fluorophosphate.

Evaluating the number of adherent bacteria, the number of vital bacteria was significantly decreased after rinsing with amine fluoride and stannous fluoride compared to the control and the sodium fluoride group (Fig. 6). Besides, the number of dead bacteria was significantly decreased after rinsing with amine fluoride and stannous fluoride compared to the control and sodium fluoride group. Further, significantly fewer dead bacteria were detected after the rinse with amine fluoride and stannous fluoride compared to sodium fluorophosphate. Nevertheless, no significant difference was revealed by the BacLight method between the rinsing solutions amine fluoride, stannous fluoride and stannous chloride (Fig. 6).

**Figure 7:**
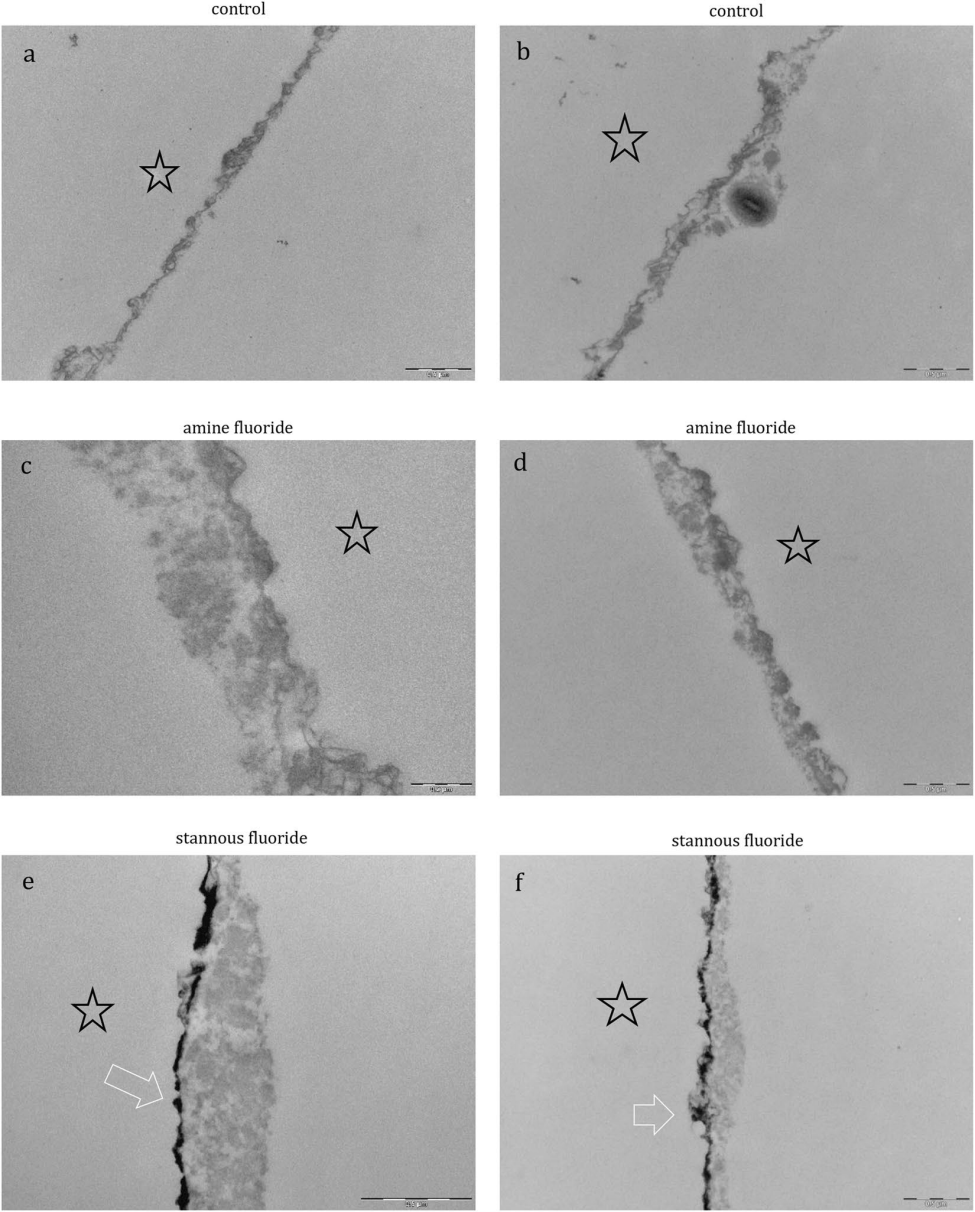
Representative TEM images visualize the pellicle´s ultrastructure. Intraoral exposure time: 8 h. All pellicles show an electron-dense basal layer. This basal layer is covered by a varying less electron-dense granular and globular layers (**a**, **b**, control). There are distinct ultrastructural differences detectable after rinsing with pure fluoride solutions (**c**–**f**) compared to the control (**a**,**b**). (**c**,**d**) Amine fluoride: An increased thickness of the less electron-dense granular and globular layer is detectable. (**e**,**f**) Stannous fluoride: A higher electron density of the basal layer with stannous precipitates (arrow) can be observed. The enamel was removed during processing of the specimens, the former enamel side is marked with an asterisk.

**Figure 8:**
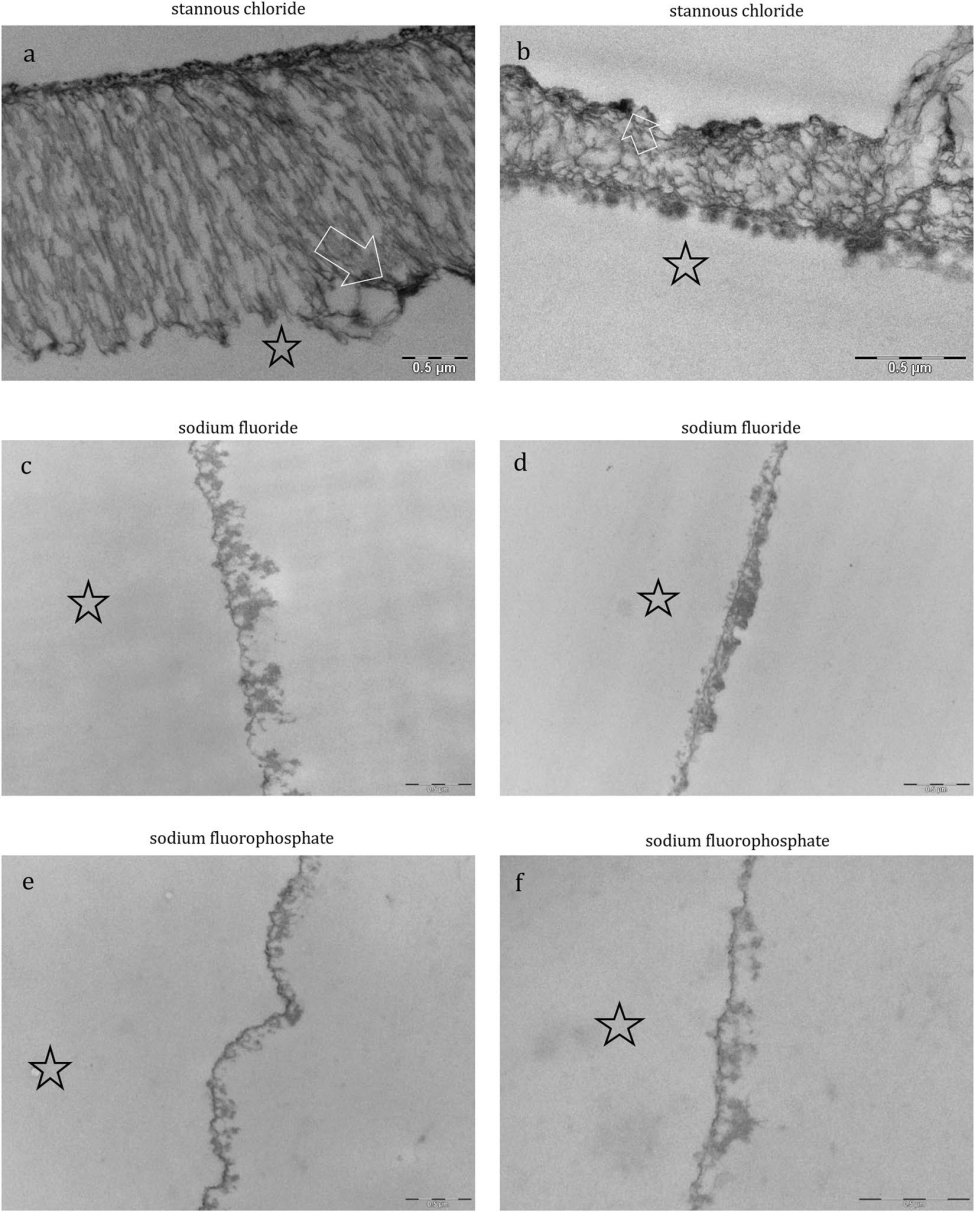
Representative TEM images visualize the pellicle´s ultrastructure. Intraoral exposure time: 8 h. All pellicles show an electron-dense basal layer. This basal layer is covered by a varying less electron-dense granular and globular layers. There are distinct ultrastructural differences detectable after rinsing with pure fluoride solutions (**c**–**f**) or stannous chloride (**a**,**b**). (**a**,**b**) Stannous chloride: A demineralization of the enamel surface took place. Infiltrated areas on the enamel surface are visible and stannous precipitates are detectable in infiltrated zones in the enamel (arrow). (**c**,**d**) Sodium fluoride: The accumulation of an electron-dense basal layer and a more inhomogenous granular layer is visible after rinsing with sodium fluoride. (**e**,**f**) Sodium fluorophosphate: A thin, electron-dense basal layer with a less electron-dense granular and globular layer with varying thickness is detectable. The enamel was removed during processing of the specimens, the former enamel side is marked with an asterisk.

The physiological accumulation of an electron-dense basal layer and a more inhomogenous second granular layer with less electron dense granular and globular structures has been confirmed by SEM as expected (Fig. 7a,b). After rinsing with sodium fluoride (Fig. 8c,d) and sodium fluorophosphate (Fig. 8e,f), the characteristic ultrastructure was visualized and showed no significant differences compared to the control. In contrast, distinct differences in the ultrastructure of the pellicle were visualized after rinsing with the pure amine fluoride, pure stannous fluoride and stannous chloride solutions (Figs. 7 and 8). Thereby, the pellicles’ thickness varied between the different pure fluoride rinsing solutions (Figs. 7c–f and 8c–f). Besides, after rinsing with amine fluoride (Fig. 7c,d) the pellicle showed an increased thickness of the less electron-dense granular and globular second layer and was denser compared to the control, sodium fluoride (Fig. 8c,d) and sodium fluorophosphate (Fig. 8e,f). But most interestingly, rinsing with stannous ions containing solutions (Figs. 7e,f and 8a,b) appeared to notably affect the characteristic ultrastructural appearance of the pellicle. After the application of stannous fluoride, the basal layer of the pellicle showed a higher electron density. In contrast, rinsing with stannous chloride (Fig. 8a,b) lead to demineralization of the enamel surface. As a consequence, infiltrated areas with stannous ions were visible (Fig. 8a,b) and stannous precipitates were detectable (Fig. 8a,b). Further, the pellicle ultrastructure showed an increased thickness compared to all other tested solutions after rinsing with amine fluoride and stannous fluoride (Fig. 7b–d).

EDX analysis was performed after the 1-min rinse of stannous fluoride and stannous chloride and the oral exposure time of 8 h. Thereby, stannous ions were detected in the pellicle layer.

## Discussion

A decline of severity and prevalence of carious lesions has been attributed to the application of fluorides^13^. Nevertheless, caries is still a global problem for the oral health systems of industrialized countries. The inhibitory effect of customary available fluoride mouthrinses against oral biofilms and their antibacterial effect has thereby been the focus of many previous studies^14,15^. However, to the authors’ best knowledge, pure fluoride substances have not been tested concerning initial bioadhesion and the modification of the pellicle´s ultrastructure under *in situ* conditions.

Screening the literature, there were suggestions that the pellicle retaines fluorides at the tooth surface^16^. Amine fluoride and stannous fluoride are assumed to have a high affinity towards hydroxyapatite surfaces and the pellicle layer, respectively. In this context, it was shown that stannous ions (Sn^2+^) in stannous fluoride (SnF_2_) have antibacterial properties^17^. These properties are the result of the inhibition of bacterial enzymes responsible for the glucose transport and bacterial metabolism^18^. The antimicrobial properties are not transient^19^ due to the very good substantivity of dental hard tissues, especially in dental biofilms^20^. In addition, Sn^2+^ ions inhibit MMPs (matrix metalloproteinases)^21^. The stannous ion as a compound of stannous fluoride or stannous chloride forms a superficial layer on the dental hard tissues^20^. Thereby, the precipitation of a calcium fluoride like product, and a stannous phosphate can bee shown^22^. Thereby, proteins from the saliva adhere to these resultant products^22^. This creates a good foundation for pellicle formation^23^. These findings are in good accordance with the results of this study. The accumulated stannous ions in the pellicle were confirmed by EDX analysis after rinsing with stannous fluoride and stannous chloride in the present study. This important finding was approved by TEM. After rinsing with stannous fluoride and stannous chloride, the incorporation of stannous ions was visible as an electron dense structure inside the pellicle layers and after rinsing with stannous chloride in the enamel surface as well. Rykke *et al*.^22^ have reported that they collected about twice as much pellicle material from the buccal surfaces of 24 stannous fluoride-treated-teeth compared to native surfaces *in vivo*^22^. Moreover, Tinanoff *et al*.^12^ detected an increase of pellicle thickness after the treatment with stannous fluoride by SEM^12^. Even this finding was affirmed in the present study. After visualization with SEM, a thicker electron dense morphology of the pellicle was detected after rinsing with pure stannous fluoride and stannous chloride (Figs. 7c,d and 8b–d). The different composition and thickness of the pellicle formed after the application of stannous fluoride seem to have an influence on the adherence of bacterial species and biofilm formation. The *In vitro-*study of Algarni confirms this assumption^24^. They examined the effect of stannous and fluoride ions on the pellicle proteome. Thereby, a higher incidence of proteins was detected after the treatment with stannous fluorides. They concluded that pellicle proteins have an impact on the precipitation of CaF_2_ reaction products on the tooth surface after the treatment with fluorides. Further, they increase the efficacy of a fluoride treatment. Overall, after the stannous fluoride treatment a higher abundance of mucins (MUC7, MUC5B), albumin and carbonic anhydrase was detected. These mucins can improve the synergy to other proteins like amylase^24,25^. This enhances the efficacy of the modified pellicle against initial bioadhesion on the tooth surface. In addition, the transient antimicrobial properties were proven by the DAPI and BacLight method after 8 h oral exposition to the oral cavity *in situ*. These results reassure the assumption that rinsing with stannous ions containing solutions leads to a retention of the stannous ions inside the pellicle layers. Hence, the pellicle can serve as a reservoir for the antibacterial stannous ions and the antibacterial, antiadherent properties of the pellicle are increased. Most interestingly, rinsing with stannous fluoride and stannous chloride reduced significantly the adherence of bacteria to the enamel surface without distinct differences between these two solutions (DAPI).

The available literature^12,19,21^ devoted to this topic gives no information about this effect. Instead, examining the antibacterial effects of stannous chloride, the accessable studies evaluate commercially availabe products that contain stannous chloride. Thereby, the most frequent combination is stannous chloride with sodium fluoride^12,15^. Accordingly, antibacterial effects were not completely attributable to stannous chloride or sodium fluoride. Nevertheless, it can be pointed out clearly, that pure stannous fluoride and stannous chloride have antiadherent effects and the stannous ions provide this effect irrespective of the combination of the tin ion with fluoride or chloride. In addition, a modified pellicle ultrastructure with thicker basal layer and thicker less electron dense granular and globular layer was detected after rinsing with the stannous ions containing solutions (Fig. 8). This observation leads to the assumption that protective properties of the pellicle are improved by stannous fluoride and chloride. Thereby, the stannous ions play an important role. The cariostatic effect of Sn^2+^ can mainly be attributed to two of its characteristics. These include antimicrobial properties and a high affinity to apatite surfaces. Oppermann and Johansen^18^ assumed that the antimicrobial activity is caused by an inhibition of microbial enzymes, which are involved in the transport, and the metabolism of glucose in bacterial cells. In addition, the acid production of biofilms is inhibited too. These antimicrobial properties are not temporary due to the high substantivity of Sn^2+^ to the tooth surface and dental biofilms^20^. Previous studies on dental erosion have already shown, that rinsing with stannous chloride and stannous fluoride leads to a precipitation of stannous ions at the tooth surface^11^. This layer maintains even after embedding in citric acid (pH 2.3). Some previous publications assume a potential molecular interaction between stannous ions and components of the pellicle layer^17^. In contrast to monovalent sodium ions, the influence of bivalent stannous ions leads to a cross-linking and an increase in adsorption of specific proteins to the pellicle layer. As a result, an increase in pellicle thickness can be detected in TEM. This includes salivary proteins like mucins and albumin. It is well known, that an increase in pellicle thickness can be associated with a decreased number of adherent bacteria to the tooth surface. In addition, the ratio of dead and vital bacteria can also be influenced. The present study has demonstrated these correlations. It could therefore be concluded that rinsing with stannous fluoride and stannous chloride leads to an increase of the pellicle thickness, resulting in a decrease of bacterial adhesion and bacterial viability.

Amine fluoride is a positively charged, antimicrobial substance with anti-plaque capacity^26^. In a previous *In-situ-*study with an amine fluoride containing mouthrinse, the potential accumulation of the amine fluorides could not be approved (EDX)^7^ and no antiadherent effect could be confirmed^7^. Adopting the same experimental set-up as Hannig *et al*.^7^ described in their *in situ* study, the present study detected as well no significant reducing effect of bacterial adherence *in situ* with DAPI. Nevertheless, as confirmed in the present study with the BacLight viability kit, biofilm viability decreased significantly after amine fluoride treatment^26^. This reduction of vital bacterial cells is mostly attributable to the electrostatic charges of the positively charged amine fluoride and the anionic bacterial cell surfaces^26^. These results underline the fact that pure amine fluoride solutions can have a significant effect on bacterial viability.

In order to obtain similar preconditions in the present study, the fluoride concentration was always adjusted to 500 ppm. As a consequence, the sample weight and measured pH-value differed among the used experimental fluoride solutions. Thereby, the most effective pure mouthrinse solutions were amine fluoride, stannous fluoride and stannous chloride, respectively. However, they displayed the lowest pH-values among the tested solutions (Fig. 1).

Interestingly, after rinsing with stannous chloride, zones of infiltration were identified in the enamel (TEM, Fig. 8). Thereby, a demineralization of the enamel surface took place. Infiltrated, etched areas on the enamel surface are visible and stannous precipitates are detectable in the infiltrated zones of the superficial enamel (Fig. 8, arrow). They can be interpreted as demineralization of the tooth surface due to the low pH of the stannous chloride rinsing solution (Fig. 1). These zones of infiltration show a demineralized, etched enamel surface filled with proteinaceous structures. In the literature, these areas are also known as subsurface pellicle and it is assumed that they have a barrier function, avoiding a further demineralization of the enamel surface^27^. Wei and Forbes already showed such an effect in their study^28^. They examined the effect of 10% stannous fluoride to the enamel surface (3 molars). Thereby, the penetration of the stannous ions was around 20 µm after 30 seconds embedding time. After these 30 seconds a demineralization of the enamel was observed and the authors of this publication described this area as an irregular zone II enamel interface configuration. Recapitulating the images from this study, Wei and Forbes have already observed the formation of a subsurface pellicle and the precipitation of stannous ions at the enamel surface after rinsing with a stannous fluoride solution in 1972^28^.

In the present study, these areas of infiltration were not detectable after rinsing with amine fluoride although both solutions had similar pH-values. Consequently, the fluoride component in stannous fluoride or the higher pH-value might be the reason that prevented the enamel surface from the demineralization event. It would certainly be very interesting to examine the antiadherent properties of stannous chloride at a higher pH-value. Thereby, demineralization could be prevented and the anticariogenic effect could still maintain.

The fluoridation of acidic agents like amine fluoride (pH 3.6) and stannous fluoride (pH 4.5) lead to changes on the hydroxyapatite surface. Thereby, acidic conditions induce complex chemical changes in the structure and compounds like Ca(OH)_2_, fluoride apatite and CaF_2_ are formed^29–31^. It was reported stannous solutions can precipitate at pH levels of 4.5 form precipitates and they can lead to dull feelings on the tooth surfaces and astringency^32^. The twelve participants of the present study did not express such feelings but stannous precipitates were detected by TEM in the ultrastructure of the pellicle after rinsing with stannous fluoride and stannous chloride.

In addition, it was shown that a higher pH of fluoride solutions is associated with lower efficiency^32^. This could be an explanation for the lower efficacy of the sodium fluoride and sodium fluorophosphate solutions in the present study. Further experiments are necessary to detect if the pH value has an influence on the bacteria-reducing effect of sodium fluoride and sodium fluorophosphate.

The present experiments on initial bacterial adherence were accomplished on bovine enamel slabs. An experimental set-up maintained in the majority of earlier studies on *in situ*^7^ and *in vitro*^8^ pellicle formation and initial bioadhesion. Thereby, the application of the fluorides was performed with a solution to ensure that mechanical brushing with a toothbrush had no additional effect. A recent study showed that an prolonged application time doesn’t lead to a thicker surface layer or an increased number of pellicle bound fluoride ions^33^. Therefore, the used application time of the fluorides was 1 min in the present study.

The use of human teeth is limited in *in situ* studies. They are difficult to obtain in higher quantities due to the degree of destruction caused by extended carious lesions leading to the extraction of the specific teeth. Thereby, it is difficult to yield a specific sample size and homogeneity of the sample surface^34^. Accordingly, alternative substrates came into focus. Different analysing methods have shown that bovine enamel mostly resembles human enamel compared to other animal species (human versus bovine, porcine and ovine)^34^. A recent study of Pelá *et al*.^35^ compared the pellicle proteome based on human and bovine enamel slabs under *in situ* conditions. Thereby, the majority of proteins were similar. The authors assume that a reason might be the similar structure of the inorganic components^35^. Therefore, human enamel can be substituted by bovine enamel when examining pellicle formation and bioadhesion under *in situ* conditions^35^. Comparing the studies on pellicle formation and investigations regarding the effect of different agents on initial bioadhesion *in situ*, all available comparative studies were conducted on bovine enamel slabs^7^. Accordingly, bovine enamel slabs were selected for the present *in situ* study.

In summary, the null hypothesis has been rejected as in the present analysis significant antiadherent effects of stannous containing solutions on the initial bacterial colonization were found. After rinsing with pure SnF_2_ and pure SnCl_2_ considerable antiadherent effects and alterations of the pellicle ultrastructure were detected. Further studies investigating the effects of pure fluorides and stannous chloride on *in vivo* biofilm formation are necessary to give more information.

## Material and Methods

### Subjects

A number of 12 caries-inactive subjects participated in the present study (Fig. 1). They were all non-smokers and showed no signs of caries or periodontitis in the last two years. Prior to the start of the study, these volunteers had given informed, written consent. An experienced dentist carried out visual oral examination. The study was conducted at the department of operative dentistry at the Dresden university hospital, Germany. The ethics committees of the Dresden (Vote: EK 275092012) University checked and approved the study design. Thereby, all research was performed in accordance with relevant guidelines/regulations. Informed written consent was obtained from all participants.

The aim of this experimental clinical *in situ* study was to compare the anti-adhesive effect of pure concentration-adjusted fluoride solutions, and one stannous-containing solution, regarding initial bacterial colonization, glucan formation and modification of the ultrastructure of the pellicle.

### Specimens

Cylindrical enamel slabs (diameter 5 mm, 19.63 mm surface area, 1.5 mm height) were gained from the facial surfaces of bovine incisors of two-year old cattle (BSE-negative animals). Slabs with structural alterations of the enamel were excluded from the study. The surfaces of the enamel slabs were polished with wet grinding abrasive paper (400–4000 grit) and the smear layer was removed by ultrasonication (us) with NaOCl (3%) for 3 min^36^. After double washing in distilled water for 5 min (us), the slabs were disinfected in ethanol (70%) for 30 min. Afterwards, they were washed in distilled water again^37,38^. Before oral exposition, the enamel slabs were stored in 4 °C aqua dest. for 24 h to form a hydration layer^37–39^.

### Tested pure solutions

Concentration-adjusted (to 500 ppm) pure experimental fluoride solutions were produced. Thereby, the the solution volume was 100 ml. Sodium fluoride (Ferdinand Kreutzer Sabamühle GmbH, Nürnberg, Germany), sodium fluorophosphates (Omya Schweiz AG, Oftringen, Switzerland), amine fluoride (Permafluor) 33% (Permcos GmbH, Arisdorf, Switzerland), stannous fluoride (Honeywell Specialty Chemicals GmbH, Seelze, Germany), and stannous chloride dihydrate (Honeywell Specialty Chemicals GmbH, Seelze, Germany) were tested in the present study. Thereby, the natural pH of the solutions was maintained during the experiments (Fig. 1): amine fluoride (pH 3.6), stannous fluoride (pH 4.5), stannous chloride (pH 3.5), sodium fluoride (pH 7.1) and sodium fluorophosphate (pH 6.6). Nonfluoridated tap water served as a reference. Between the different examination days, a two-day wash out period was performed.

### Pellicle formation

For *in situ* pellicle formation individual upper jaw splints were customized from stainless-steel clamps and polymethylmethacrylate^40,41^. Cavities for the insertion of the enamel slabs were prepared on the buccal sites of the premolars and 1^st^ molar on both sites (n = 8/splint). The fixation of the enamel specimens was performed with polyvinyl siloxane impression material (Aquasil, Denstply De-Trey, Konstanz, Germany). One hour before wearing the splints, the subjects brushed their teeth without toothpaste, flossing was optional, and eating was not allowed. To allow pellicle formation and initial bacterial colonization on the specimens‘ surfaces, the splints were carried intraorally for 1 min followed by a 1-min rinse with the specific test solution (tap water, sodium fluoride, sodium fluorophosphate, amine fluoride, stannous fluoride and stannous chloride), and 8 h of intraoral exposure (Fig. 1).

### Total bacterial count (DAPI) and glucan formation (Con A)

DAPI staining was conducted as described previously^36^. DAPI (4′,6-diamidino-2-phenylindole) stains DNA unspecifically by binding to the AT-rich regions of double stranded DNA^42^. Upon binding to DNA, the DAPI molecule fluoresces intensely. First, the samples were rinsed with sodium chloride. For staining, the samples were covered with 1 ml DAPI solution (Merck, Darmstadt, Germany) in a dark chamber. After 15 min the DAPI solution was removed, and the samples were covered with methanol for 4 min. In the following, the specimens were dried at room temperature and coated with Citifluor (Citifluor Ltd., London, UK) and analysed by epifluorescence microscopy (Axioskop II, Zeiss, Oberkochen, Germany). The initial biofilms were analysed at 1000-fold magnification using the light filter for DAPI (BP 365, FT 395, LP 397). The number of cells observed in ten randomized microscopic ocular grid fields per sample was counted. The area of ocular grid (0.0156 mm^2^) allowed calculating the numbers of bacteria per square centimeter. DAPI staining was combined with concanavalin A (Invitrogen, Molecular probes, Darmstadt, Germany) for visualization of glucan formation. The stock solution was 5 mg/ml Alexa Fluor 594 conjugate in 0.1 M NaH_2_PO_4_ buffer, pH 8.3. The stock solution was stored at −20 °C. The working solution was a 10-μl stock solution in 490 μl PBS (1 mM CaCl_2_, 1 mM MnCl_2_, 1 mM MgCl_2_).

### BacLightTM viability assay

The LIVE/DEAD® BacLight™ Bacterial Viability Kit (Invitrogen, Molecular probes, Darmstadt, Germany) adopts two nucleic acid stains: green-fluorescent SYTO^®^ 9 stain and red-fluorescent propidium iodide stain^43^. The BacLight kit was used for staining of enamel samples exposed to the oral fluids for visualization of vital and dead bacteria in the adherent state. Similar amounts of component A (Syto9 1.67 mM/propidium iodide 1.67 mM, 300 μl DMSO) and B (Syto9 dye 1.67 mM/propidium iodide 18.3 mM, 300 μl DMSO) were mixed; 2 μl were added to 1 ml of saline solution. The samples were rinsed with sodium chloride. Afterwards the enamel slabs were incubated with this solution in a dark chamber for 10 min. Finally, the samples were rinsed with saline solution and evaluated immediately with a fluorescence microscope using the FDA filter and the ethidium bromide filter^7^.

### Ultrastructural analysis of the *in situ* formed pellicle (TEM)

The pellicle-covered enamel slabs were fixed in 2.5% glutaraldehyde/1.5% formaldehyde for 2 h. Postfixation took place in 1% osmium tetroxide for 2 h. The specimens were dehydrated in an ascending series of alcohol and embedded in Araldite CY 212 (Plano, Wetzlar, Germany). After decalcification in 1 M HCl re-embedding was accomplished with Araldite. Ultrathin sections of the pellicle layer were cut with a diamond knife, mounted on pioloform-coated copper grids and contrasted with uranyl acetate and lead citrate. TEM analysis of the pellicle´s ultrastructure was performed at 30,000–100,000 fold magnification in a TEM TECNAI 12 Biotwin (FEI Eindhoven, The Netherlands).

### Energy-dispersive X-Ray spectroscopy (EDX)

EDX-analysis was performed after application of stannous fluoride and stannous chloride *in situ* (Fig. 1). The analysis was carried out on these block-face specimens with a XL 30 ESEM (philips, Eindhoven, Netherlands) with an EDX system (Phoenix, EDAX INC., Mahwah, USA) in order to detect stannous ions immobilized in the pellicle layer.

### Statistics

Statistical evaluation of the number of adherent bacteria (DAPI and BacLight) as well as the number of glucans was carried out using the Kruskal-Wallis and the Mann–Whitney U-test (p < 0.05)^44^. Afterwards, a Bonferroni-Holm correction took place. The used Software was SPSS 21.0 (IBM, Ehningen, Germany). Regarding the statistical sample size analysis (Fig. 1) the power of the study is 80%. In the present study the following null hypothesis is tested with DAPI, ConA, BacLight and TEM: No significant effects will be found between the applied pure fluoride rinsing solutions and the control group.

## Data Availability

The datasets generated and analysed during the current study are available from the corresponding author on reasonable request.
