# Author Correction: Influence of pure fluorides and stannous ions on the initial bacterial colonization *in situ*

**DOI:** 10.1038/s41598-020-62064-1

**Published:** 2020-03-25

**Authors:** Jasmin Kirsch, Matthias Hannig, Pia Winkel, Sabine Basche, Birgit Leis, Norbert Pütz, Anna Kensche, Christian Hannig

**Affiliations:** 1 Clinic of Operative Dentistry, Medical Faculty Carl Gustav Carus, Technische Universität Dresden, Fetscherstraße 74, D-01307 Dresden, Germany; 2 Clinic of Operative Dentistry, Periodontology and Preventive Dentistry, University Hospital, Saarland University, Building 73, D-66421 Homburg, Saar Germany

Correction to: *Scientific Reports* 10.1038/s41598-019-55083-0, published online 06 December 2019

The original version of this Article contained errors in the spelling of the authors Jasmin Kirsch, Matthias Hannig, Pia Winkel, Sabine Basche, Birgit Leis, Norbert Pütz, Anna Kensche, Christian Hannig which were incorrectly given as Kirsch Jasmin, Hannig Matthias, Winkel Pia, Basche Sabine, Leis Birgit, Pütz Norbert, Kensche Anna & Hannig Christian respectively.

Additionally, this Article contained typographical errors.

In the Results section,

“The bovine specimens were analysed with DAPI, BacLight, SEM and EDX-analysis (Fig. 1).”

now reads:

“The bovine specimens were analysed with DAPI, BacLight, TEM and EDX-analysis (Fig. 1).”

“The physiological accumulation of an electron-dense basal layer and a more inhomogenous second granular layer with less electron dense granular and globular structures has been confirmed by SEM as expected (Fig. 7a,b).”

now reads:

“The physiological accumulation of an electron-dense basal layer and a more inhomogenous second granular layer with less electron dense granular and globular structures has been confirmed by TEM as expected (Fig. 7a,b).”

Finally, in the Discussion section,

“Moreover, Tinanoff *et al*.^12^ detected an increase of pellicle thickness after the treatment with stannous fluoride by SEM^12^. Even this finding was affirmed in the present study. After visualization with SEM, a thicker electron dense morphology of the pellicle was detected after rinsing with pure stannous fluoride and stannous chloride (Figs. 7c,d and 8b–d).”

now reads:

“Moreover, Tinanoff *et al*.^12^ detected an increase of pellicle thickness after the treatment with stannous fluoride by TEM^12^. Even this finding was affirmed in the present study. After visualization with TEM, a thicker electron dense morphology of the pellicle was detected after rinsing with pure stannous fluoride and stannous chloride (Figs. 7c,d and 8b–d).”

These errors have now been corrected in the PDF and HTML versions of the Article.

